# Room-temperature control and electrical readout of individual nitrogen-vacancy nuclear spins

**DOI:** 10.1038/s41467-021-24494-x

**Published:** 2021-07-20

**Authors:** Michal Gulka, Daniel Wirtitsch, Viktor Ivády, Jelle Vodnik, Jaroslav Hruby, Goele Magchiels, Emilie Bourgeois, Adam Gali, Michael Trupke, Milos Nesladek

**Affiliations:** 1 Institute for Materials Research (IMO), Hasselt University, Diepenbeek, Belgium; 2 Faculty of Biomedical Engineering, Czech Technical University in Prague, Kladno, Czechia; 3 Institute of Organic Chemistry and Biochemistry of the Czech Academy of Sciences, Prague, Czechia; 4 Faculty of Physics, University of Vienna, Vienna, Austria; 5 Wigner Research Centre for Physics, Budapest, Hungary; 6 Department of Physics, Chemistry and Biology, Linkoping University, Linköping, Sweden; 7 IMOMEC Division, IMEC, Diepenbeek, Belgium; 8 Department of Atomic Physics, Budapest University of Technology and Economics, Budapest, Hungary

**Keywords:** Electronic properties and materials, Quantum information, Optical properties of diamond, Qubits

## Abstract

Nuclear spins in semiconductors are leading candidates for future quantum technologies, including quantum computation, communication, and sensing. Nuclear spins in diamond are particularly attractive due to their long coherence time. With the nitrogen-vacancy (NV) centre, such nuclear qubits benefit from an auxiliary electronic qubit, which, at cryogenic temperatures, enables probabilistic entanglement mediated optically by photonic links. Here, we demonstrate a concept of a microelectronic quantum device at ambient conditions using diamond as wide bandgap semiconductor. The basic quantum processor unit – a single ^14^N nuclear spin coupled to the NV electron – is read photoelectrically and thus operates in a manner compatible with nanoscale electronics. The underlying theory provides the key ingredients for photoelectric quantum gate operations and readout of nuclear qubit registers. This demonstration is, therefore, a step towards diamond quantum devices with a readout area limited by inter-electrode distance rather than by the diffraction limit. Such scalability could enable the development of electronic quantum processors based on the dipolar interaction of spin-qubits placed at nanoscopic proximity.

## Abstract (web summary)

Nuclear spins in diamond are promising for applications in quantum technologies due to their long coherence times. Here, the authors demonstrate a scalable electrical readout of individual intrinsic ^14^N nuclear spins in diamond, mediated by hyperfine coupling to electron spin of the NV center, as a step towards room-temperature nanoscale diamond quantum devices.

## Introduction

The development of a physical platform for practical quantum computation is among the most compelling scientific and technological goals of quantum technologies^1^. Although many recent achievements based on superconducting circuits or trapped ions have been made, semiconductor spin qubit systems may offer significantly wider electronic scalability and nanoscale device integration. Nuclear spins pose a viable alternative for quantum computation and, due to their exceptional isolation from the environment and resulting long coherence times, they were employed in some of the early demonstrations of functional quantum algorithms^2,3^. Nuclear spins in solids can additionally act as quantum memories^4–17^, enhancing the sensitivity of quantum sensors and quantum repeaters. The negatively charged nitrogen-vacancy (NV) centre in diamond is one of the most attractive solid-state qubit platforms^18–20^, for which high fidelity single- and two-qubit NV electron and nuclear qubit gates^21^, as well as quantum error correction protocols^22^, have been demonstrated. Consequently, surrounding ^13^C (nuclear spin-1/2) and ^14^N or ^15^N (nuclear spin 1 or 1/2) atoms can be engineered in the diamond lattice for this purpose, and control of up to 27 nuclear spins via single NV electron spin has been achieved^23^. In addition, it was demonstrated that entanglement can be distributed probabilistically between two NV electron spin qubit nodes using a spin–photon interface^24,25^, combining optical detection of magnetic resonance (ODMR) with photon manipulation techniques. In this scenario, nuclear spins can serve as data qubits connected and read out by an electron spin of the nearby NV centre operating at low temperature (~4 K)^26^. However, for the development of scalable quantum computation in diamond the probabilistic nature of this concept, as well as limited entanglement rates on the order of 40 Hz reached so-far^27^, represent a complex problem yet to be solved.

Here we demonstrate an approach for a quantum technology platform operating at room-temperature via electrically-read electron-nuclear spin gates using a diamond electronic chip. This approach can significantly reduce technological hurdles for the realisation of future diamond quantum devices. In recent works^28–33^, we have developed a technique in which the spin state of a coherently controlled NV electron spin is read out electrically by photoelectric detection of magnetic resonance (PDMR). Unlike optical readout, for which spatial resolution is usually diffraction-limited, the spatial resolution achievable by PDMR for individual NVs is solely determined by the electrode size. The demonstrated increase of NV production yield via co-doping^34^, or very recent achievements of nanometre precision implanting^35^, opened the way towards nearly deterministic NV generation. With the reduction in the PDMR interrogation area to the nanoscale regime, the consequent individual readout of the NV spins, placed at dipole-dipole interaction distance^14^, with such implanting technologies would provide a significant step towards small entangled qubit arrays.

PDMR is based on the spin state-dependent photoionization of the NV centre, mediated by charge state conversion^32^. The application of an external electric field accelerates the photo-generated charge carriers towards the electrodes fabricated on the diamond surface. Thus, the NV spin state can be determined by directly measuring the photocurrent without the need for photon collection, even for single NV centres^31^. PDMR features several additional benefits for spin readout since, in contrast to conventional optical detection schemes, it does not suffer from the excited state saturation at high incoming photon fluxes^31^. In the case of photocurrent, the saturation is instead given by the charge carrier recombination lifetime in diamond, which is several orders of magnitude longer than the excited state lifetime^31^. In combination with high electron collection efficiency, PDMR readout may therefore enable shot noise reduction due to significantly higher electron detection rates compared to photon counting^32^.

To advance towards nanoscale NV qubit systems, we demonstrate the electrical readout of its basic element, a two-qubit electron-nuclear spin system. Although the readout of large ensembles of nuclear spins has recently been achieved by using electrically-detected electron-nuclear double resonance (EDENDOR)^36^, the electrical readout of a single NV nuclear spin has remained elusive. In this work, we overcome this challenge and achieve room-temperature photoelectric readout of the single intrinsic ^14^N nuclear spin of the NV centre, mediated by the electron spin, making use of the spin polarisation (and spin state detection) near the excited-state level anti-crossing (ESLAC). We are able to detect nuclear magnetic resonance spectra and coherent spin rotations with high contrast, even for the long intervals between laser excitation pulses required for nuclear spin manipulation. We also provide evidence that the ESLAC conditions enable highly efficient MW-free protocols for the electrical detection of the nuclear spin, which are of interest for low-jitter quantum gates. To describe the experimentally measured photoelectric detection rates, we develop a theoretical model using the Lindblad master equation. The model provides an underlying theory for photoelectric readout techniques and includes charge state transitions, time-dependent spin polarisation and charge carrier readout at the ESLAC. Future implementations of more complex schemes, such as the inclusion of nearby ^13^C nuclei, can now be envisaged aiming towards scalable quantum hardware for quantum computation and sensing.

## Results

### Single NV photoelectrical detection and imaging

**Fig. 1:**
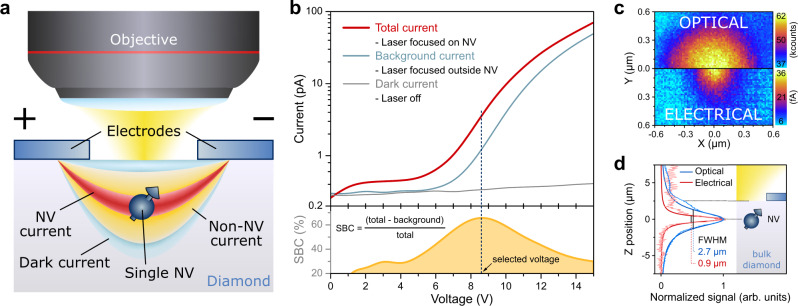
Electrical detection of single NV centre. **a** Schematic of the PDMR chip with the single NV centre used for the measurements. A yellow-green 561 nm laser is focused between the contacts (with an inter-electrode distance of 3.5 µm) using a microscope objective in air (N.A. = 0.95). The resulting currents are measured versus the bias voltage applied to the electrodes. We identify three types of currents: dark current—not related to the laser illumination, non-NV photocurrent—laser-induced photocurrent not originating from the NV centre, NV photocurrent—current from the two-photon ionization of the single NV centre. **b** Current–voltage characteristic curves for laser off (grey, dark current), for laser (4 mW) focused away from the single NV centre (cyan, background current from dark current and non-NV photocurrent) and for laser (4 mW) focused on the single NV centre (red, total current from dark current, non-NV photocurrent, and NV photocurrent). The bias voltage for the photocurrent measurements was set to 8.6 V as determined from the maximum signal-to-background contrast (SBC) calculated from the background and total current (dark yellow). **c**, **d** Simultaneous optical and electrical imaging of the single NV centre (laser power 6 mW). **c** XY map showing the size comparison of the same NV centre for the two detection methods. **d** Z scan of the NV. Darker curves are the Lorentzian fits of the experimental data points, FWHM stands for full width at half maximum calculated from the fitted data.

Throughout the experiments, a commercial IIa high-pressure high-temperature (HPHT) diamond (New Diamond Technology, <10 ppb background nitrogen), with intrinsic single NV defects, was used. To collect charge carriers under bias voltage, coplanar interdigitated contacts with a 3.5 µm gap were fabricated on the diamond surface by means of optical lithography. The photocurrent was pre-amplified and then recorded via lock-in detection^30^ (see Supplementary Fig. 1 for the detailed schematic of photoelectric measurement principle). We stochastically allocated a few individual NV centres between the electrodes and selected those ~2.5 µm below the diamond surface. To obtain optimal qubit operational parameters, we first measured the DC current–voltage (*I*–*V*) characteristics, in which we identified three main current components (see Fig. 1a): (i) NV photocurrent—resulting from two-photon ionization of the single NV centre, (ii) non-NV photocurrent—produced by photoionization of other diamond defects and (iii) dark current—present after application of the voltage between electrodes without laser illumination. The sum of the dark and non-NV currents, denoted further as a background current, was determined from measurements with the laser beam focused between contacts, but off the NV centre. The measured DC *I*–*V* characteristics (see Fig. 1b) show nonlinear characteristics, as discussed previously^31^. After having calculated the signal-to-background contrast (SBC), we set the bias voltage to the optimal 8.6 V, corresponding to the highest measured NV SBC (>65%). However, in some practical applications, an increase in bias voltage might prove beneficial to increase signal acquisition at the cost of SBC.

In order to reduce the photocurrent contribution induced by photoionization of substitutional nitrogen (P1 centres, photoionization onset ~550 nm)^37,38^ frequently present in diamond crystals, we used a yellow-green 561 nm (2.21 eV) laser instead of the commonly applied green excitation (532 nm). Optimal laser powers for photoelectric imaging of the single NV centre have been previously identified to be between 2 and 4 mW^31^. To enable detection of low average photocurrents imposed by the prolonged pulse sequences needed for nuclear spin manipulation (discussed below), higher laser powers (4–6 mW) are applied here in order to increase the NV-generated photocurrent. Interestingly, even at these comparatively high laser powers, unlike photoluminescence, photocurrent does not saturate (see Supplementary Fig. 2 for saturation scans) and a higher S/N ratio is reached. However, further increase of laser power would lead to a reduction of the NV magnetic resonance contrast unless the laser readout pulses are made correspondingly shorter^32^. The selected NV centre was imaged by scanning the focused laser beam with simultaneous electrical and optical detection to allow for a direct comparison between the two readout methods (see Fig. 1c). Due to the two-photon nature of NV ionization, as well as the Gaussian beam shape after the focussing objective, electrical imaging has been shown to significantly improve both the spatial resolution and the imaging contrast^31^. Here, we observe an even more substantial improvement in resolution in all three dimensions, with a threefold reduction of the axial size for electrical imaging (see Fig. 1d).

### Photoelectric readout principle at ESLAC

**Fig. 2:**
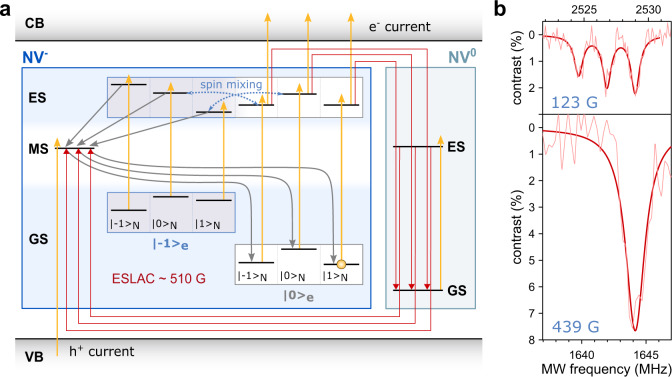
PDMR detection at ESLAC. **a** Schematic describing the photoelectric readout principle at ESLAC. Only transitions responsible for the PDMR contrast between the different electron and nuclear spin states are visualized. The |m_s_〉 = |+1〉 state, which was not probed in these experiments, is omitted for clarity. Under the application of the magnetic field (~510 G for ESLAC), the NV^−^ centre ground state (GS) energy levels |m_s_〉 = |−1〉 and |m_s_〉 = |0〉 are well separated (~1.5 GHz), whereas the excited state (ES) becomes nearly degenerate resulting in spin mixing between the states with the equivalent total spin projection quantum number. The spin mixing combined with the electron spin polarisation to |m_s_〉 = |0〉 through the metastable state (MS) [grey arrows], results in the spin polarisation to the |m_s_〉 = |0〉 electron and |m_I_〉 = |+1〉 nuclear spin state. The yellow arrows depict optical transitions induced by the application of the yellow-green laser. As can be seen, the |m_s_〉 = |0〉 spin sublevels in the ES are more likely to be excited by the second photon and contribute to the photocurrent by promoting the NV electron to the diamond conduction band (CB). When this happens, the negatively charged NV^−^ centre is converted to NV^0^ centre (red arrows). The back-conversion is possible by another two-photon process while preserving the nuclear spin orientation. First, the NV^0^ centre is excited to the ES and subsequently, an electron is promoted from the valence band (VB) to the vacated orbital of NV^0^, leading to the formation of NV^−^ centre. In this process the NV^−^ |m_s_〉 = |0〉 ground states efficiently repolarise. **b** Pulsed PDMR measurements of the NV nuclear (^14^N) and electron (m_s_ = −1) spin hyperfine interaction for different magnetic fields showing nuclear spin polarisation close to the ESLAC (experimental conditions for measurement at 123 G—1500 ns laser pulse of 4 mW power, 1100 ns long MW π-pulse; for measurement at 439 G—1000 ns laser pulse of 6 mW power, 400 ns long MW π-pulse).

A schematic of the model describing the photoelectric readout principle at the excited-state level anti-crossing (ESLAC) is depicted in Fig. 2a, including charge transitions of the NV centre. The full photoionization model is discussed in more detail in the section ‘Modelling’. To achieve photoelectric readout of a single nuclear spin using the NV centre electron spin as an ancilla, we first polarise the ^14^N nuclear spin to the |m_I_〉 = |+1〉 state^39–42^. When an external magnetic field of ~510 G (approaching the ESLAC) is aligned with the NV axis, optical pumping polarises the nuclear spin into the |m_I_〉 = |+1〉 state (spin polarisation of >98%^39^) due to the spin state mixing and simultaneously initializes the electron spin into |m_s_〉 = |0〉^39–41^. This allows us to directly perform the coherent nuclear spin control and sensitively obtain the spin state via photocurrent measurements, as the electron photoionization probability does depend on the electron-nuclear spin states. In order to electrically detect nuclear hyperfine interactions, a pulsed lock-in envelope readout technique^30^ was employed (see ‘Methods’ for more detail). In this detection method, readout laser pulses are solely applied during the high-state of the low-frequency envelope duty cycle, while the pulsed microwave (MW) driving is performed continuously. The rising edge of the frequency envelope is then used to trigger the lock-in amplifier. As can be seen in Fig. 2b, PDMR clearly resolves the hyperfine spectrum of a single nuclear spin close to the ESLAC.

### Modelling

To provide a theoretical framework describing spin and photoelectric transitions, we model the NV centre using the Lindblad master equation. The model allows us to calculate the resulting spin contrast and compare PMDR and ODMR readouts at various experimental conditions. We utilize the following formalism to calculate the time dependence of the density matrix

1 $$\dot{\rho }=-\frac{i}{{\hslash}}\left[H,\rho \right]+\mathop{\sum}\limits_{k}{\varGamma }_{k}\left({L}_{k}\rho {L}_{k}^{\dagger }-\frac{1}{2}\left\{{L}_{k}{L}_{k}^{\dagger },\rho \right\}\right),$$

where *H* and *ρ* are the Hamiltonian and the density matrix of the system and *L*_*k*_ are Lindblad jump operators carrying out non-unitary transitions with rates ${\varGamma }_{k}$. The model includes five electronic states, namely the ground state, optical excited state, and the singlet shelving state in the negative charge state as well as ground and excited states in the neutral charge state of the NV centre. In the simulations, we use 13 Lindblad operators that describe the transitions between different electronic states (rates can be found in Supplementary Table 1). The spin Hamiltonian includes electron, nuclear and hyperfine spin interactions in the triplet ground and excited state of the NV^−^. The model is further described in the ‘Methods’ section and is detailed in Supplementary Note 6.

**Fig. 3:**
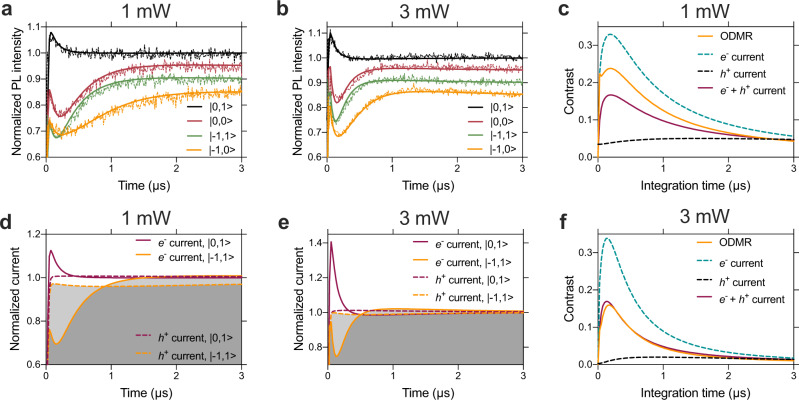
Photon and charge carrier emission dynamics and intrinsic spin contrast. **a**, **b** The experimental (thin) and theoretical (bold) time traces of the normalized PL intensity upon turning on the laser excitation pulse of 1 and 3 mW power, respectively, for various initial electron and nuclear spin states. For a better visibility, the curves corresponding to |0,0〉, |−1,1〉 and |−1,0〉 initial states are shifted down by 0.05, 0.1 and 0.15, respectively. To prepare different initial spin states in the experiment, RF, MW0 and MW1 π-pulses were used (see Fig. 4a). The theoretical results closely follow the experimental curves. **d**, **e** The time traces of the simulated electron currents (solid lines) and hole currents (dashed lines) for 1 and 3 mW laser power, respectively, for |0,1〉 and |−1,1〉 initial spin states. The curves are normalized to the steady-state electron current obtained for the |0,1〉 initial spin state. The areas below the electron and hole currents (grey areas under the solid and dashed lines) are integrated to the same value to ensure the charge neutrality constraint of the photoionization cycle. The difference between the solid (dashed) curves provides the spin contrast of the electron (hole) current. **c**, **f** The theoretical ODMR contrast and the contrast of the electron-only current, the hole-only current, and the total current (PDMR) as a function of integration time for 1 and 3 mW excitation power, respectively. In the case of ODMR and PDMR, the contribution of the experimental background signal is taken into consideration as well.

To determine the power-dependent ionization rates, we record time traces of the optical signal starting from different initial spin states of the NV^−^. These time traces reveal important details of the charge state dynamics of the NV centre (see Supplementary Note 6) and allow for validation of the model, which reproduces the experimental PL curves (see Fig. 3a, b). In addition, it allows us to predict the time-dependent electron and hole currents, which are not accessible in the current experimental setup. The time dependence of the normalized electron current closely follows the PL time traces (see Fig. 3d, e). We attribute this similarity in the dynamics of the photon emission and photoionization to the predominant contribution from the occupation of the NV^−^ excited states for both processes. On the other hand, we note that the amplitudes of the spin-dependent PL and photocurrent signals show a different power dependence. Besides electrons, photo-emitted holes additionally contribute to the final spin contrast, prolonging it on longer timescales. Regarding the power dependence of the contrast, we obtained a larger ODMR than PDMR contrast at 1 mW and comparable ODMR and PDMR contrasts at 3 mW (see Fig. 3c, f). While this trend is similar to what has been observed in our experiments, our microscopic model on the isolated NV centre cannot quantitatively account for all the power-dependent observations. Therefore, we conclude that the residual deviation between the experimental and theoretical ODMR and PDMR contrasts are not related to the intrinsic properties of the NV centre, but rather to its lattice environment and further investigations into the charge state dynamics are of high interest. Unlike emitted photons, charge carrier traps and recombination centres may significantly affect electron and hole currents and their ratio. Indeed, nonlinear recombination processes, which may occur more substantially at high laser powers, can enhance the PDMR contrast (see Supplementary Note 7). The fundamental differences in the detection of ODMR and PDMR signals may provide a path to a highly efficient PMDR quantum detection with improved contrast by optimizing material design and device fabrication aspects.

### Coherent electronic and nuclear qubit rotations

**Fig. 4:**
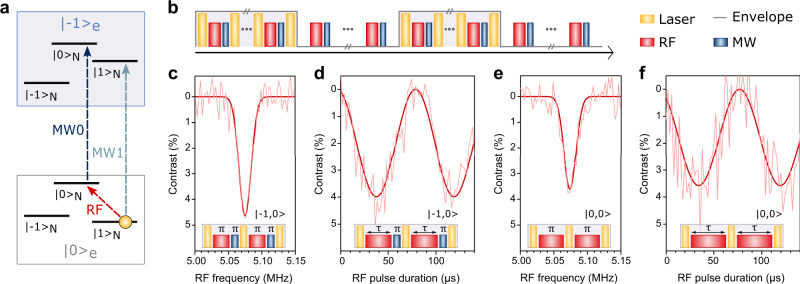
Electrical readout of individual nuclear spin. **a** Schematic of the NV ground state hyperfine energy-level structure of the |m_s_〉 = |0〉 and |m_s_〉 = |−1〉 states depicting the resonant frequencies of the probed transitions (RF: radiofrequency, MW0: resonant microwave frequency to selectively excite the electron spin in |m_I_〉 = |0〉, MW1: resonant microwave frequency to selectively excite the electron spin in |m_I_〉 = |+1〉). **b** Scheme of the envelope pulse train designed for electrical readout of a single nuclear spin using the lock-in detection technique. Here, the lock-in amplifier is triggered by the on/off envelope modulation of the laser pulses. **c** Electrically-detected RF resonant frequency of the |0,+1〉 and |0,0〉 transitions of the single ^14^N nuclear spin measured at 439 G. Inset shows the pulse sequence used, consisting of RF and MW0 π-pulses. **d** Corresponding electrically-detected Rabi oscillations of the single nuclear spin with the pulse sequence shown in the inset. **e** Electrically-detected RF resonant frequency of the |0,+1〉 and |0,0〉 transitions of the single ^14^N nuclear spin without electron spin manipulation measured at 439 G. Inset shows the pulse sequence used consisting of RF π-pulses. **f** Corresponding electrically-detected Rabi oscillations of the single nuclear spin with the pulse sequence shown in the inset (experimental conditions: 4000 ns laser pulse of 6 mW power, 400 ns long MW π-pulse, 1 W RF power).

To coherently drive and read out the nuclear spin at the ESLAC, we used a set of RF pulses, applied to the nuclear spin, combined with MW-assisted electron spin readout. First, using the relatively high nuclear spin polarisation at 439 G, we probed the transition between |0,+1〉 and |−1,0〉 sublevels (see Fig. 4a). To include the radiofrequency (RF) driving necessary to manipulate the nuclear spin, the envelope lock-in readout technique^30^ was modified (see Fig. 4b). The pulse sequence consisted of a laser pulse for spin polarisation and photocurrent readout, followed by an RF pulse to drive the nuclear spin from |m_I_〉 = |+1〉 state to |m_I_〉 = |0〉 state and a 400 ns long MW π-pulse (MW0), set to selectively swap the |m_I_〉 = |0〉 electron spin from the |m_s_〉 = |0〉 state to the |m_s_〉 = |−1〉 state (see Fig. 4a). In this measurement, similarly to optical detection, the nuclear spin contrast is measured through the signal amplitudes of the particular spin states. To measure the resonance frequency of the nuclear spin, an RF pulse with a length corresponding to a π-rotation on the Bloch sphere was applied while sweeping its frequency (see Fig. 4c). The laser power was kept at 6 mW with a pulse duration of 4 µs where single nuclear spin PDMR showed a contrast close to 5%. To demonstrate nuclear Rabi oscillations, the resonant RF pulse length was increased stepwise, while the distance between laser pulses was kept constant to conserve the same number of pulses per envelope duty cycle. The resulting electrically-detected Rabi nutation (see Fig. 4d) maintained a high contrast even for long RF pulse durations (up to 140 µs). We note that, depending on the duration of the nuclear spin rotation, these operations already correspond to an entangling CNOT gate, for which the electron and nucleus reach their highest level of entanglement at the π/2-point (~18 µs, in Fig. 4). While we do not characterize their performance here, such gates can reach high fidelity in NV centres, even at room temperature^43^.

Unlike optical detection, where a short window (typically 300 ns) at the start of the readout pulse is recorded, the photocurrent is integrated during the entire pulse sequence, as to date current pre-amplifiers do not allow for sufficiently fast (>10 MHz) gating with high (10^12^) current amplification at room temperature. Photocurrent integration, therefore, reduces the maximum detected spin contrast, as both the electron and nuclear spins are initialized within the first ~0.5 µs of the laser pulse^44^. Even though the detected contrast decreases using longer laser pulses and higher laser powers (see Fig. 3c, f), due to intrinsic characteristics of the readout, the PDMR contrast is maintained over prolonged time periods due to the hole current as devised from the theoretical modelling above (note that the optical curves in Fig. 3a, b are shifted for clarity). A further improvement of the detected PDMR contrast is given by the slower nuclear spin polarisation of the |m_I_〉 = |−1〉 and the |m_I_〉 = |0〉 states (due to the spin mixing at ESLAC), which spans 3× and 2× longer (respectively) than the |m_I_〉 = |+1〉 state^40^. Furthermore, the lower technical noise, achieved using the small electrode architecture, and lock-in readout technique permits the high nuclear PDMR spin contrast reached in our measurement. Development of fast current preamplifiers will enable further significant improvement of the measured spin contrast.

The scheme presented above is realised by both MW (electron) and RF (nuclear) spin driving. However, in many applications, it might be advantageous to use MW-free readout (e.g. to reduce additional heating in MW absorbing environments and/or to avoid the MW resonant frequency jitter in prolonged measurements). Interestingly, the spin mixing between the nuclear and electron spins near the ESLAC permits to achieve such MW-free readout via the reverse polarisation of the electron spin by the nuclear spin^40,42^. To demonstrate this possibility, we modified the detection sequence excluding the MW driving pulses. For the MW-free detection scenario, Fig. 4e, f shows the nuclear resonances and Rabi nutations, respectively. Surprisingly, we achieved a contrast similar to the MW-assisted readout using the MW-free detection, indicating that the electron spin can be repolarised by the nuclear spin very efficiently, leading to a low loss in the spin contrast. The measurements depicted in Fig. 4c–f were additionally repeated at a higher B-field of 500 G, yielding similar results (see Supplementary Fig. 6), demonstrating that the spin mixing at ~439 G is sufficiently strong for the PDMR of a single nuclear spin. The differences in the spin contrast have also been theoretically modelled and are further discussed in Supplementary Note 7.

## Discussion

The experimental results presented in this paper represent the first demonstration of the photoelectrical detection of a single nuclear spin at room-temperature, achieved using two-qubit spin gates applied to the electron spin and the intrinsic ^14^N nuclear spin of the NV centre. The developed theoretical model allows for a precise description of spin-dependent photocurrent and its time dependency at the excited-state level anti-crossing (ESLAC) and can be used for a highly efficient spin control and readout. PDMR keeps a high spin contrast, even for prolonged sequences used for nuclear spin manipulation. The demonstration of a basic unit of a nuclear spin coupled to the NV electron is a first step towards the development of more complex, electrically-read gates, employing the benefits of long nuclear spin coherence in technological, solid-state electronic realizations. By providing a method for site-selective readout of electron spins, that further interact with adjacent nuclear spins, this work attempts to clear an important hurdle towards dipolarly interacted spin-qubits nodes placed in nanoscopic proximity, and construction of diamond quantum devices with semiconductor scalability. Future work is needed to develop a site-selective individual electron spin polarisation at such short node distances. In this direction, the wavelength-selective resonant spin polarisation might be used in combination with the Stark shift at low temperature^45^. For such control, the nanoscopic electrodes used as a field gate can provide controllable wavelength shift, or even manipulation with the NV charge state^46^. In addition, magnetic gradients can provide high-fidelity spin control at the nanoscale^47^. The rich toolbox of control methods for the NV centre therefore provides a ground for the development of electronic diamond quantum processors and scalable diamond quantum devices.

## Methods

### Experimental setup

The measurements were carried out using a custom-built setup allowing simultaneous optical and electrical detection (see Supplementary Fig. 1a). For laser excitation, a yellow-green 561 nm gem laser from Laser Quantum was used with the continuous-wave power measured before the objective ranging between 4 and 6 mW. The pulses were created using acousto-optic modulator and the beam was focused on the sample using air objective (×40, N.A. 0.95). As-purchased IIa electronic grade [111] high-pressure high-temperature diamond sample from New Diamond Technology was equipped with interdigitated contacts (3.5 µm gap) and a MW line (see Supplementary Fig. 1b, c) by means of optical lithography using sputtering deposition (20 nm layer of titanium covered with 100 nm layer of aluminium). The electrodes and the MW line were wire-bonded to the printed circuit board tracks. The bias electric field (~2.5 × 10^4^ V cm^−1^) was applied between the electrodes and the photocurrent was collected, after preamplification (×10^12^, Standford Research SR570 pre-amplifier), by a lock-in amplifier (Standford Research SR850) referenced to the 7 Hz envelope frequency.

Due to the fact that currently available photocurrent detection electronics do not offer sufficiently fast gating options for the desired electrical current amplification range, pulsed photocurrent detection is commonly averaged over the entire pulse sequence. The comparatively long sequences required for nuclear spin manipulation (a factor of about 1000 compared to electron spin manipulation) can, however, result in a decreased averaged photocurrent and an excessive amount of DC noise accumulation during this period. Therefore, to circumvent this problem, we use a low-frequency modulation that envelopes the fast laser pulses and allows for frequency-filtered readout of an averaged photocurrent in a slower pulse^30^. This method, however, currently does not allow to provide time-dependent photocurrent detection within one laser excitation pulse. Such improvement relies on the development of fast, low-noise current preamplifiers and switches, which would further enhance the pulsed PDMR spin contrast.

### Nuclear PDMR

For nuclear spin polarisation near the ESLAC, a neodymium magnet was mounted on a holder (five degrees of freedom) and aligned to the NV axis. Due to the high lateral and axial resolution of the photoelectric detection, the sample drift during the measurements is more prominent in the electrical signal. Refocusing was therefore performed between PDMR scans by maximizing the pulsed photocurrent signal in all three spatial directions. For the Rabi measurements, additional non-resonant RF (4 MHz) was applied in the sequence segment where resonant RF and MW pulses were off, in order to provide a constant heat load on the sample for different resonant RF pulse durations.

### Hyperfine model and photocurrent simulations

In order to simulate the PDMR and ODMR contrast, we utilize a five-electronic-state model, see Supplementary Note 6. Each electronic state includes nuclear spin and electron spin degrees of freedoms. Time-dependent rates of transitions between certain electronic states are calculated from the population of the states, obtained from the diagonal elements of the time-dependent density matrix, and corresponding transition rates. Photon emission rate is determined from the calculated excited state to ground state transition rates, while charge carrier injection rate is obtained from the rate of the photo-induced charge state transitions of the NV centre.

To incorporate the hyperfine interactions at the ESLAC we considered the spin Hamiltonians of the excited state (ES) and ground state (GS) of the negatively charged NV centre, which can be written as

2 $${H}_{{\rm{ES}}({\rm{GS}})}^{\left(-1\right)}={D}_{{\rm{ES}}({\rm{GS}})}\left({S}_{z}^{2}-\frac{2}{3}\right)+{g}_{e}{\mu }_{B}{\bf{BS}}{\boldsymbol{+}}{\bf{S}}{A}_{{\rm{ES}}\left({GS}\right)}{\bf{I}}+P\left({I}_{z}^{2}-\frac{2}{3}\right)-{g}_{14N}{\mu }_{N}{\bf{BI}},$$

where ${\bf{S}}$ and ${\bf{I}}$ are the electron and the ^14^N nuclear spin operator vectors, ${D}_{{\rm{ES}}}=1.42$ GHz is the zero-field-splitting and ${A}_{{\rm{ES}}}$ is the hyperfine tensor with eigenvalues ${A}_{\perp }=27.45$ MHz and ${A}_{\parallel }=41.42$ MHz in the excited state, ${D}_{{\rm{GS}}}$ and ${A}_{{\rm{GS}}}$ are the zero-field splitting and the hyperfine tensor in the ground state^48^, respectively, and $P=-5.01$ MHz is the quadrupole splitting^49^. For the singlet shelving state, we used the following expression

3 $${H}_{{\rm{SS}}}^{\left(-1\right)}=P\left({I}_{z}^{2}-\frac{2}{3}\right)-{g}_{14N}{\mu }_{N}{\bf{BI}}$$

In all electronic states, we neglected the orbital momentum degrees of freedom, which are indispensable for understanding the spin selective non-radiative decay process but affect the spin dynamics of the electronic states negligibly^16^. As the Zeeman splitting of the doublet state NV^0^ suppresses hyperfine mixing between the electron and nuclear spins, the electron spin degrees of freedom of the spin-1/2 neutral charge state can be neglected at the magnetic field values considered in our experiments. Therefore, ${H}_{{\rm{GS}}}^{\left(0\right)}={H}_{{\rm{ES}}}^{\left(0\right)}\approx {H}_{{\rm{SS}}}^{\left(-1\right)}$ was used in the simulations. Due to the opening of a large energy gap between the *m*_*s*_ = +1 and other electron spin sublevels in the ground and excited states of the negative charge state, only the most relevant *m*_*s*_ = {0,−1} spin states were included in our model.

## Supplementary information

Supplementary Information

## Data Availability

The data supporting the conclusions of this article are included within the article and in the Supplementary Information. In addition, the source data are available in figshare repository with the identifier [10.6084/m9.figshare.14748138.v1].
