# Supplementary material for: Room-temperature control and electrical readout of individual nitrogen-vacancy nuclear spins
Source: Nat Commun. 2021 Jul 20;12:4421. doi: 10.1038/s41467-021-24494-x (PMC8292375; doi:10.1038/s41467-021-24494-x)
Supplement: Supplementary file 1 — Supplementary Information [file 41467_2021_24494_MOESM1_ESM.docx]

SUPPLEMENTARY INFORMATION

**Room-temperature control and electrical readout of an individual nitrogen-vacancy nuclear spins**

**Michal Gulka^1,2,3†*^, Daniel Wirtitsch^4†^, Viktor Ivády^5,6^, Jelle Vodnik^1,7^, Jaroslav Hruby^1,7^, Goele Magchiels^1^, Emilie Bourgeois^1,7^, Adam Gali^5,8^, Michael Trupke^4^ and Milos Nesladek^1,2,7*^**

^1^Institute for Materials Research (IMO), Hasselt University, Wetenschapspark 1, B-3590 Diepenbeek, Belgium.

^2^Faculty of Biomedical Engineering, Czech Technical University in Prague, Sítná sq. 3105, 272 01, Kladno, Czechia.

^3^Institute of Organic Chemistry and Biochemistry of the Czech Academy of Sciences, 166 10 Prague, Czechia.

^4^Faculty of Physics, University of Vienna, VCQ, Boltzmanngasse 5, 1090 Vienna, Austria.

^5^Wigner Research Centre for Physics, PO. Box 49, Budapest H-1525, Hungary.

^6^Department of Physics, Chemistry and Biology, Linkoping University, SE-581 83 Linköping, Sweden

^7^IMOMEC division, IMEC, Wetenschapspark 1, B-3590 Diepenbeek, Belgium.

^8^Department of Atomic Physics, Budapest University of Technology and Economics, Budafoki út 8., H-1111, Budapest, Hungary

^†^These authors contributed equally to this work

^*^Corresponding authors, emails: [gulka.michal@gmail.com](mailto:gulka.michal@gmail.com), [milos.nesladek@uhasselt.be](mailto:milos.nesladek@uhasselt.be)

**Supplementary Note 1: Setup for electrical measurements:**

To perform our measurements, a custom-built confocal setup for photoelectric detection of magnetic resonances (PDMR) was used. In contrast to typical 520 or 532 nm continuous‑wave (CW) excitation, we employed a yellow-green 561 nm laser (gem 561, Laser Quantum) for both the optical and the photoelectrical detection. Pulsed excitation, as well as slow (7 Hz) laser modulation necessary for lock-in detection^1^, was done via an acousto-optic modulator (AOM 3200-146, EQ Photonics) and the laser beam was then focused on the sample using an air objective (40x, N.A. 0.95, Olympus). Furthermore, a bias voltage (8.6 V, equivalent to an electric field of ~ 2.5×10^4^ V·cm^-1^) was applied at the diamond contacts using a low-noise voltage source (487 Keithley) and the photocurrent was pre-amplified using a pre-amplifier (Standford Research SR570, amplification of 10^12^) and subsequently read out by a lock-in amplifier (Stanford Research SR850) referenced to the 7 Hz envelope frequency. To deliver the microwave (MW) and radiofrequency (RF) a SynthNV Signal Generator (Windfreak) and STEMLAB 125-10 (Red Pitaya) were used respectively. These signals were then pulsed using fast switches (ZASWA‑2‑50DR+, Mini-Circuits), amplified (ZHL-42+ for MW and ZX60-100VH+ for RF, both Mini-Circuits) and combined using a custom-built diplexer (TTE Filters). As a TTL source used to trigger the MW, RF switches as well as the AOM pulses, a pulse generator (Pulse Streamer 8/2, Swabian Instruments) was used. Additionally, for the time-resolved optical measurements, photons were collected using a single‑photon counting module (SPCM-AQRH-14, Excelitas Technologies) and were assigned timestamps using a streaming time-to-digital converter (Time Tagger 20, Swabian Instruments). In order to apply the external DC magnetic bias field, a stock neodymium magnet’s field was aligned with respect to the single nitrogen-vacancy (NV) axis using a custom‑built magnet holder (with 3-axes movement, rotation and 2 goniometers).


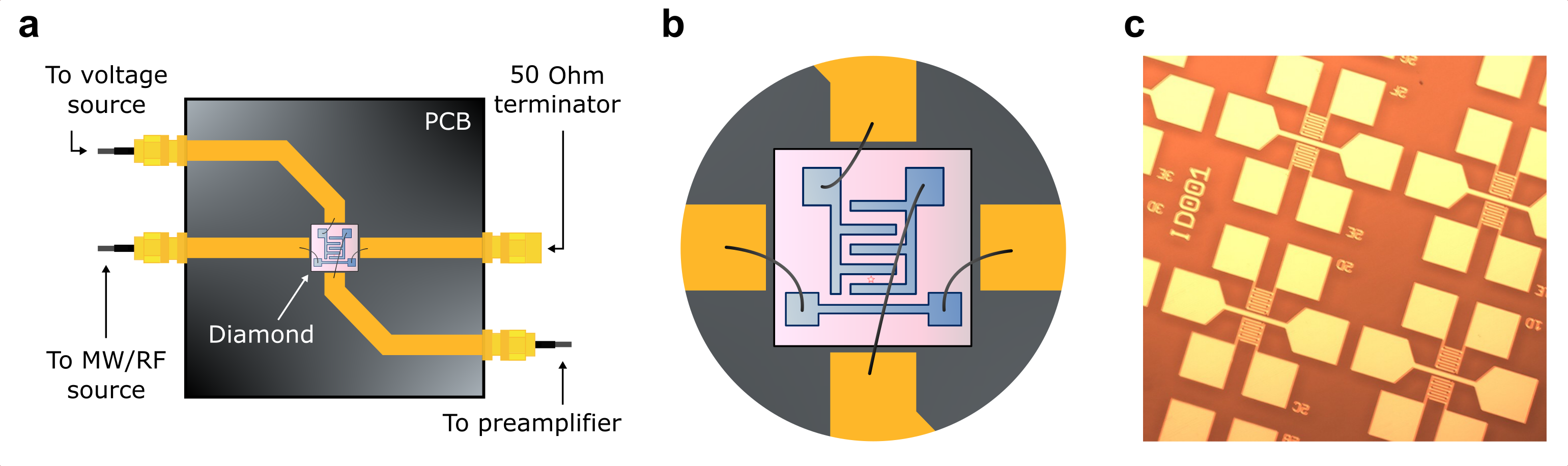


**Supplementary Figure 1 | Diamond chip for electrical detection of single NV centre**. **a**, A schematic of the printed circuit board (PCB) used for PDMR measurements. **b**, Zoom on the diamond chip connected to the PCB tracks for electrical detection and MW/RF excitation. **c**, An optical image of the deposited structures on the diamond surface.

**Supplementary Note 2: Diamond chip preparation:**

All measurements were carried out using a commercial electronic grade type-IIa diamond plates (New Diamond Technology, < 10 ppb background nitrogen) with intrinsic single NV centres. Before electrode deposition, the diamond sample was rinsed in deionized water and cleaned in a mixture of H_2_SO_4_ and KNO_3_. The sample was first placed in the ~10 ml of H_2_SO_4_ and heated to ~250-300°C. Once the H_2_SO_4_ started fuming, ~10 mg of KNO_3_ was added and the sample was kept in the mixture for ~1 hour at a constant temperature. Subsequently, the diamond was rinsed in deionized water and dried. To create ohmic bonds for electric readout, interdigitated contacts (3.5 um gap) were deposited on the cleaned diamond surface by standard optical lithography, using 20 nm layer of titanium covered with 100 nm layer of aluminium. The electrodes were then wire-bonded to the custom‑made printed circuit board (PCB) tracks using a 25 µm thick aluminium wire (see Supplementary Figure 1). SMA connectors for MW and RF driving and photocurrent readout were connected to the tracks and grounded to the common ground on the backside of the PCB. MW was applied either via a 50 μm wire or via metal tracks formed by the optical lithography on the diamond surface.

**Supplementary Note 3: Characterization of the single NV PDMR:**

In typical optical measurements such as optical detection of magnetic resonances (ODMR), applied laser powers are set below the saturation limit imposed by the value of the NV^-^ excited state. However, in these conditions photoionization during readout is usually negligible and the electron is kept mostly in the initial charge state. For PDMR, generation of free charge carriers occurs due to the two-photon ionization (optical excitation and subsequent ionization from the excited states) and, due to the short (13 ns) lifetime of the optically excited states, predominantly occurs at higher laser powers^2^. Photocurrent, unlike photoluminescence (PL) intensity, does not suffer from the NV excited state saturation behaviour even at comparatively high powers (see CW saturation scans in Supplementary Figure 2a). Saturation is instead given by the charge carrier recombination lifetime in diamond, which is several orders of magnitude longer^3^ than the excited state lifetime. Consequently, a higher S/N ratio can be reached. However, unless the laser readout pulses are made sufficiently short, laser pulses with too much power would lead to a reduction of the NV magnetic resonance contrast for pulsed PDMR experiments. For the pulsed electrical readout of the single nuclear spin, a laser power of 6 mW with a pulse duration to 4 µs was used, which enabled detection of the low average photocurrent imposed by the prolonged pulse sequences needed for nuclear spin manipulation (up to 142 µs). Since currently available photocurrent detection electronics (e.g. preamplifiers and lock-in amplifiers) do not offer sufficiently fast gating options for desired amplification range, photocurrent detection is averaged over the entire pulse sequence, hence reducing our readout contrast. Development of fast, low-noise current preamplifiers and switches might thus further enable significant improvement of the measured spin contrast for pulsed PDMR experiments.


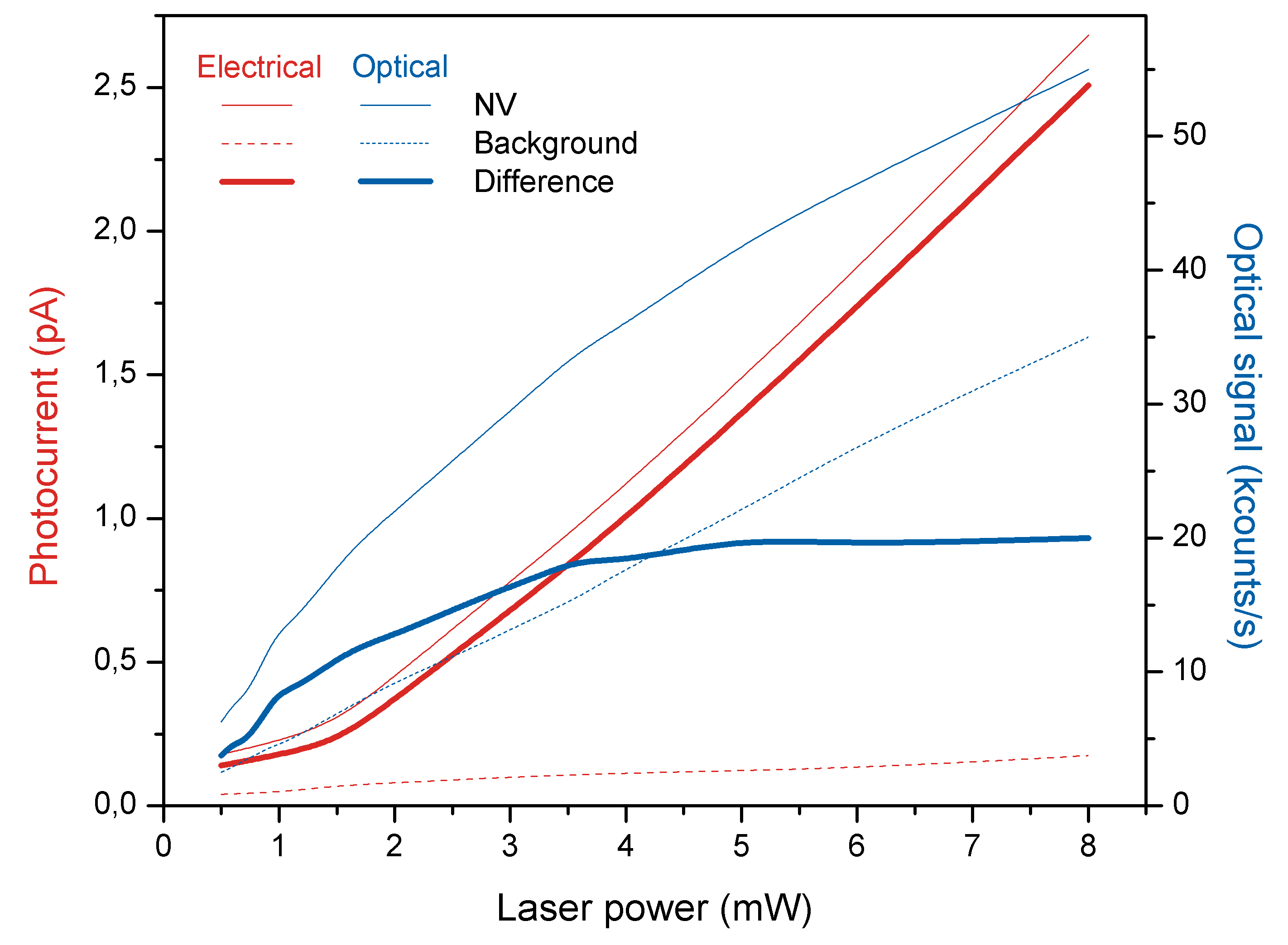

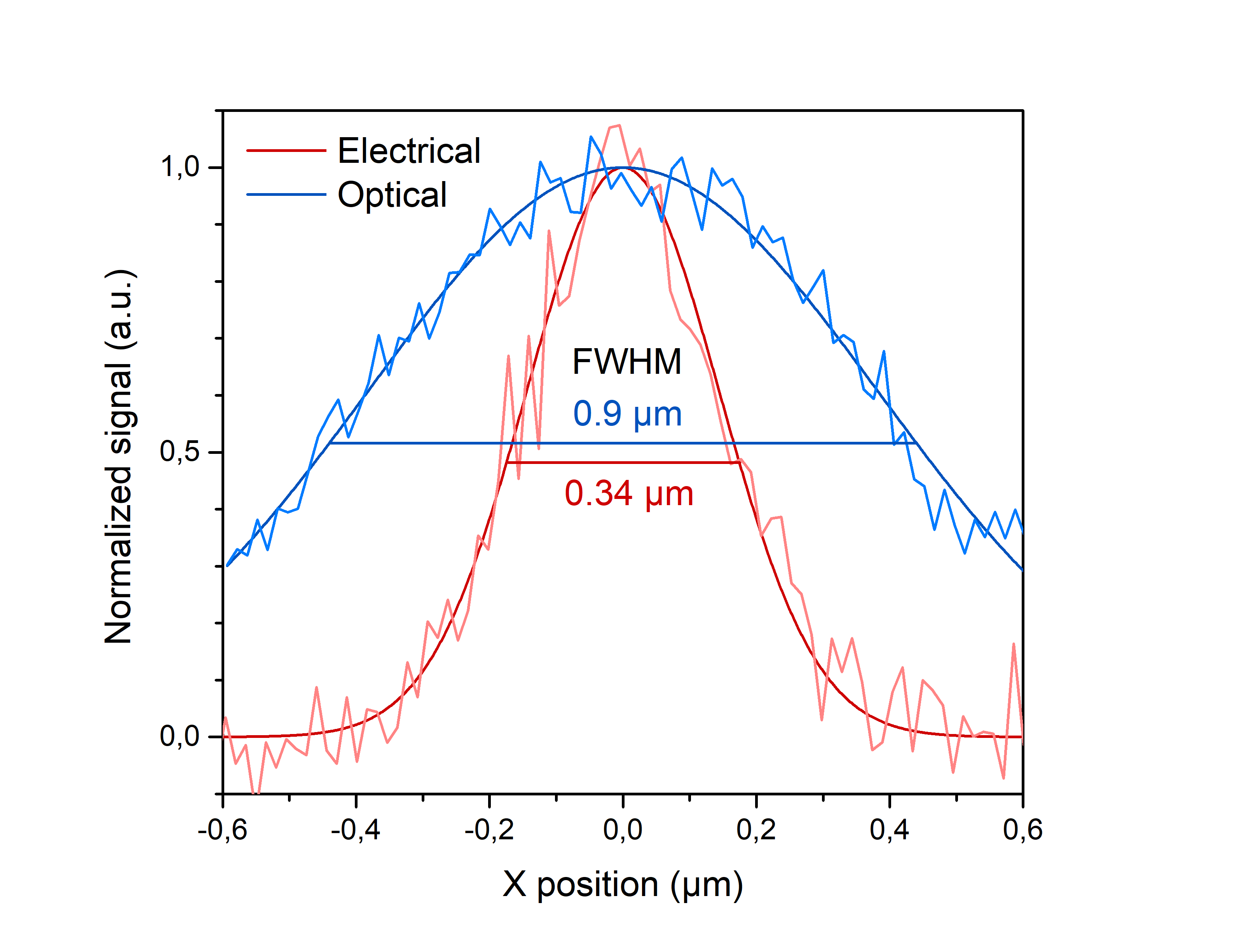


a

b

**Supplementary Figure 2 | CW saturation scans**. **a**, Electrical (red) and optical (blue) signals with respect to the CW laser power measured on (NV – thin line) and away (background – dashed line) of the NV centre in between the contacts. The thick line represents the difference between the NV and background measurements. **b**, X scan of the single NV (taken from the Figure 1c image from the main text). Darker curves are the Gaussian fits of the experimental data points for the used objective (NA 0.95).

We characterized electrical detection of single NV centres by spatial imaging using a lock-in detection technique (see Supplementary Figure 2b) and measured DC current‑voltage (I‑V) characteristics (see main text). The images are acquired with a NA 0.95 air objective, where the two-photon ionization significantly improves spatial resolution. However, shadowing of the laser beam with depth due to the relatively narrow contact spacing might have contributed to the worsened optical resolution. The device was operated at bias voltage set to the optimal 8.6 V (see main text), corresponding to the highest measured NV signal-to-background contrast (>65%). However, in some practical applications, an increase in bias voltage might prove beneficial for increasing signal acquisition rate at the cost of the signal-to-background contrast. Furthermore, it should be noted that optimal parameters might depend heavily on the nature of the observed background current, such as possible current nonlinearities (when changing laser power), as well as on instrumentation parameters like amplification noise, contact noise, etc.

**Supplementary Note 4: Spin contrast at the excited state level anti crossing:**

At the excited‑state level anti‑crossing (ESLAC) optical excitation leads to a state selective spin mixing of the NV’s electron and nuclear spin^4–6^. This mixing, together with a favourable branching from the electrons optically excited |m_s_〉 = |±1〉 states into the long‑lived singlet (MS), can effectively and efficiently initialize both electron and nuclear spin into |m_s_, m_I_ 〉 = |0, +1〉. A schematic of the described process is shown in Supplementary Figure 3. As elaborated in more detail in the theory sections, we describe photocurrent generation via photoionization from the optical excited states of the negative charge state of the NV. The neutral charge state is modelled as a two-level system from which charge recapture creates a hole which additionally attributes to the obtained photocurrent.


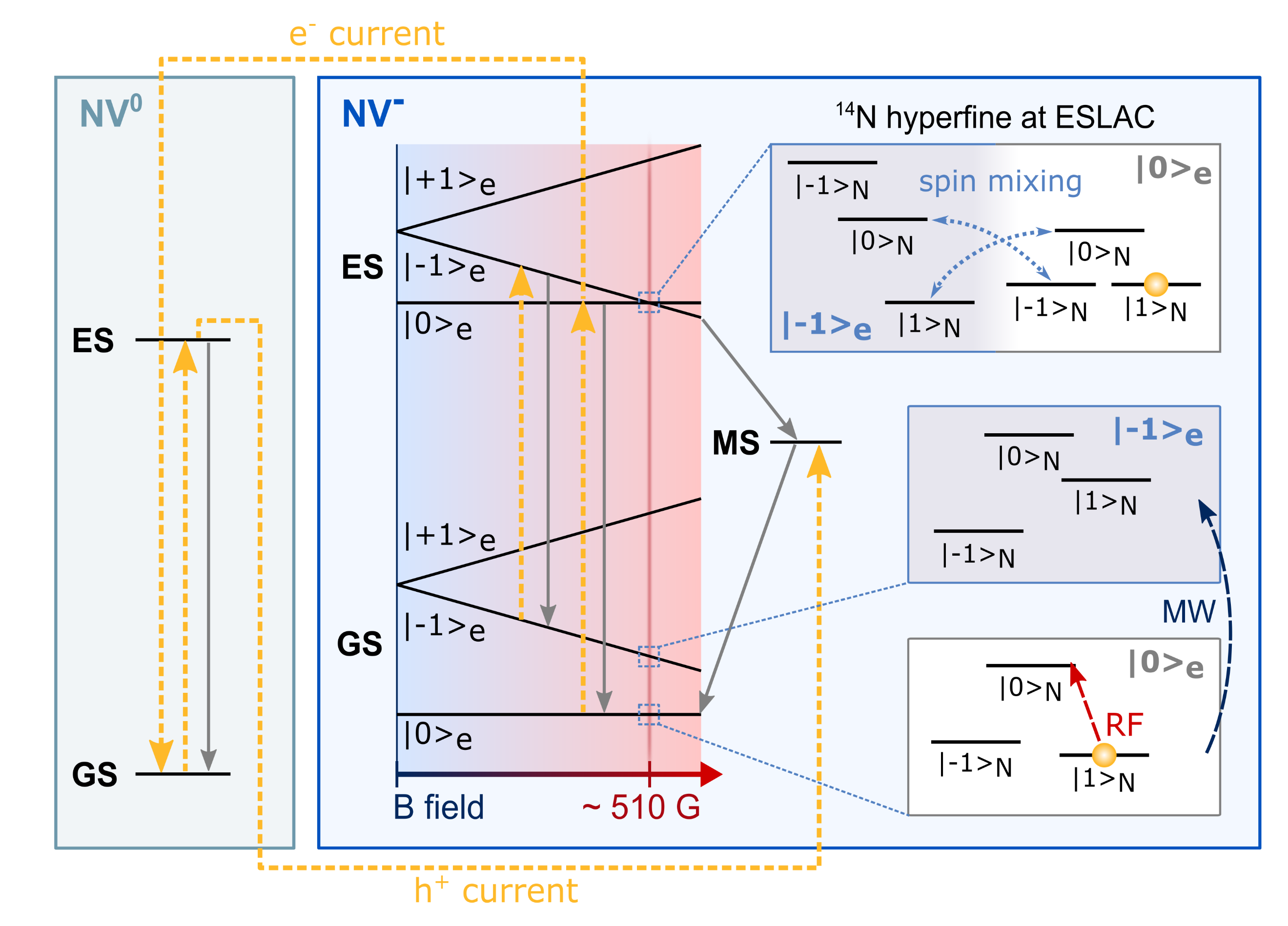


**Supplementary Figure 3 | Scheme of PDMR principle approaching ESLAC**. Some of the transitions that are not responsible for spin contrast are omitted. (GS – ground state, ES – excited state, MS – metastable state)

To validate our model parameters, and to gain insights into the complex transition dynamics, we measured the time-dependent pulsed ODMR spin contrast behaviour for different initial electron-nuclear spin states near the ESLAC. The sequence consisted of repetitive 4 µs laser pulses to achieve spin polarization and readout, and MW/RF π‑pulses for spin rotations between laser excitation. Photons were assigned time-stamps with a bin size of 10 ns and the resulting photon histograms are depicted in Supplementary Figure 4. The optical traces were measured for three different magnetic field values approaching the ESLAC and four different laser powers. From the experimental data, the time evolution of contrast (see Supplementary Figure 4) is calculated as a difference between the polarized state |0,+1〉 and probed state (i.e. |0,0〉, |‑1,+1〉 or |‑1,0〉) signals divided by the polarized state signal. Our measurements clearly indicate the possibility of microwave-free nuclear spin readout near the ESLAC as used in our measurement (see main text). Typically, nuclear spin manipulation is followed by a microwave readout pulse on the electron spin, converting population into fluorescence (blue lines). Comparison of the fluorescence traces for |0,0〉 (yellow) and |0,1〉 (red), which differ solely by the nuclear spin state, however, shows that while at low magnetic fields nuclear spin‑dependent fluorescence contrast vanishes, near the ESLAC spin flip dynamics in the optical excited state obviate the need for such a readout pulse. Therefore, the microwave‑free nuclear spin readout can be performed with a comparable spin contrast. We note that these additional dynamics should be considered when conventional nuclear spin protocols are read out via application of a final microwave pulse since, e.g., readout contrast between |0,0〉 and |-1,0〉 does not necessarily match the contrast obtained from |0,1〉 and |-1,1〉.


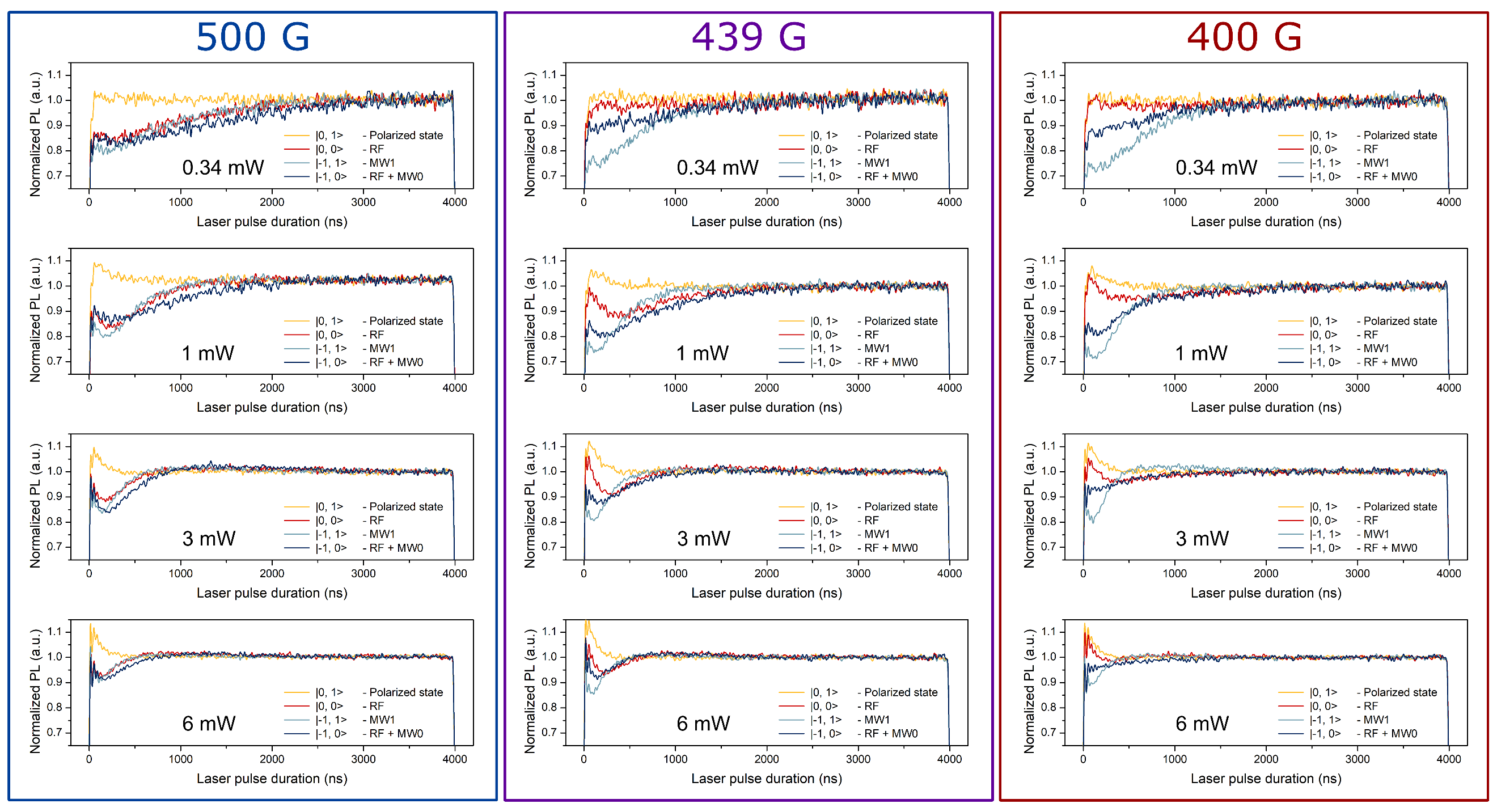


**Supplementary Figure 4 | Optical time traces for different electron‑nuclear spin states**. Recorded histograms of photon counts collected experimentally within the duration of the laser pulses for different laser powers, magnetic fields and initial states. The traces are normalized to the steady‑state photoluminescence at the end of the readout pulse. The corresponding transitions for the MW0, MW1 and RF frequencies are depicted in Main text Figure 4a.


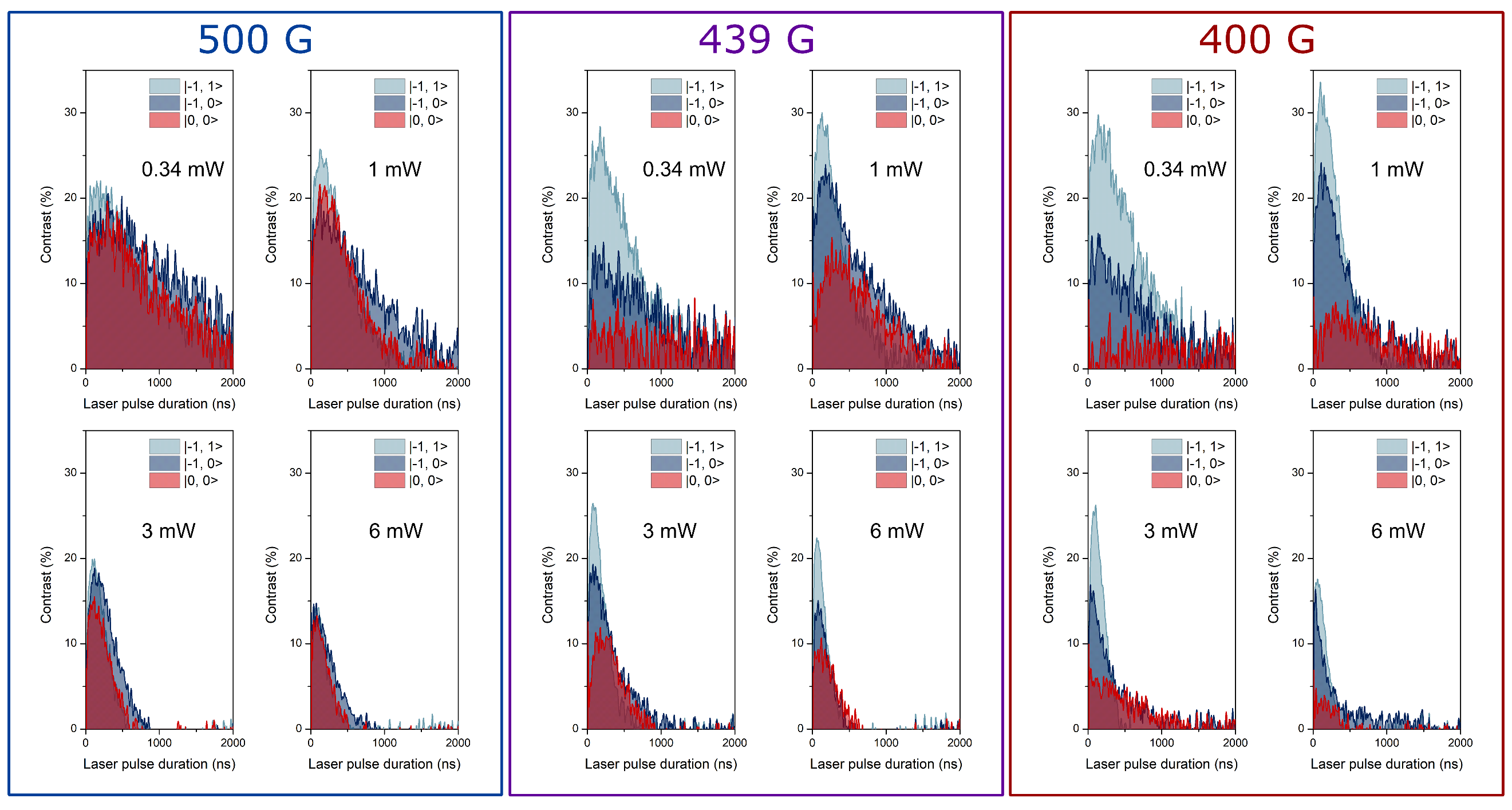


**Supplementary Figure 5 | Optical contrast evolution**. Time-dependent spin contrast for different electron‑nuclear spin states calculated from the experimental data in Supplementary Figure 4.

**Supplementary Note 5: Coherent electronic and nuclear qubit rotations**

Single nuclear spin PDMR experiments described in Figure 4 (Main text) were also carried out at 500 G. The resulting nuclear resonances and Rabi nutations are shown in Supplementary Figure 6 for comparison, where we perform both MW-free and microwave mediated photoelectric nuclear spin readout. As discussed in the previous section, we obtain almost the same readout contrast in both cases.


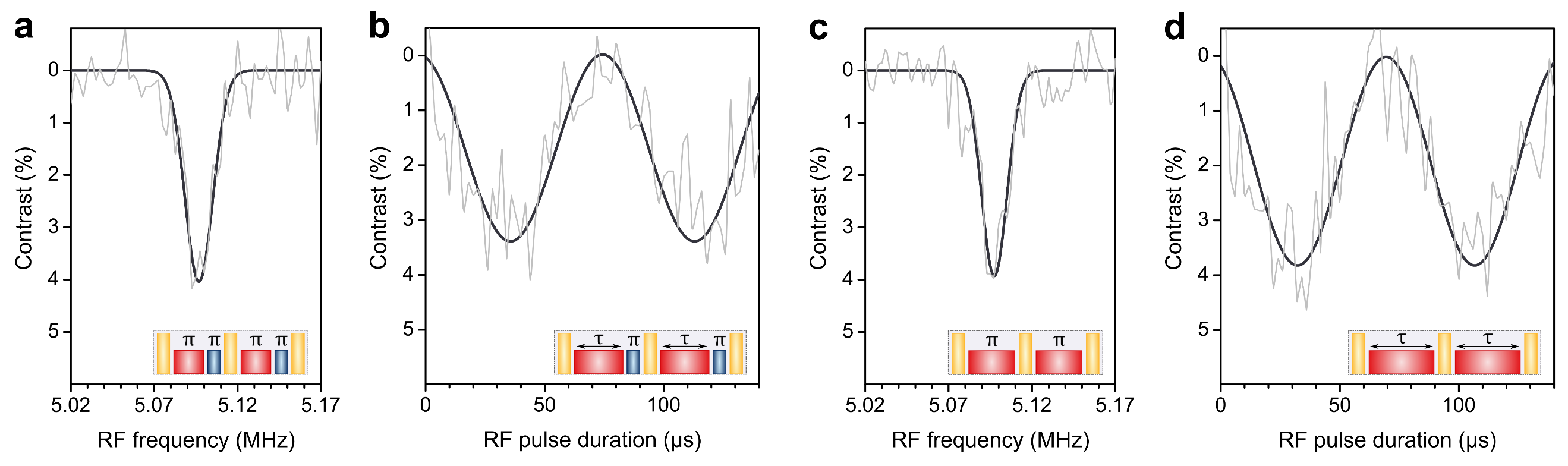


**Supplementary Figure 6 | Single nuclear spin PDMR at 500G**. **a**, Electrically‑detected RF resonant frequency of the |0,+1〉 and |0,0〉 transitions of the single ^14^N nuclear spin measured at 500 G. The inset shows the corresponding pulse sequence, consisting of RF and MW π‑pulses. **b**, Electrically‑detected Rabi oscillations of the single nuclear spin with the corresponding pulse sequence shown in the inset. **c**, Electrically‑detected RF resonance frequency of the |0,+1〉 and |0,0〉 transitions of the single ^14^N nuclear spin without MW electron spin manipulation (MW‑free detection) measured at 500 G. The inset shows the respective pulse sequence, consisting of RF π‑pulses. **f**, Electrically‑detected MW-free Rabi oscillations of the single nuclear spin with the pulse sequence shown in the inset. (Experimental conditions: 4 µs long laser pulse with 6 mW power, 400 ns MW π‑pulse, 1 W RF power).

**Supplementary Note 6: Theory of ESLAC transitions and modelling - methodology part**

The spin‑dependent optical excitation cycle of the NV centre was modelled by the Lindblad master equation:

$\dot{\rho}=-\frac{i}{\hbar}\left[ H,\rho\right]+\sum_{k} \Gamma_{k}\left( L_{k}\rho L_{k}^{\dagger}-\frac{1}{2}\left\{ L_{k}L_{k}^{\dagger},\rho\right\} \right)$, (1)

where $H$ and $\rho$ are the Hamiltonian and the density matrix of the system, respectively. $L_{k}$ are Lindblad jump operators carrying out non-unitary transitions with rates $\Gamma_{k}$. Our model includes five electronic states, as depicted in Supplementary Figure 7, in particular the ground state, the optical excited state, the singlet shelving state in the negative charge state, as well as the ground and excited neutral charge states of the NV centre. The five electronic states additionally include the electron and ^14^N nuclear spin sublevels. The Hamiltonian can be written as

$H=\left( \begin{matrix} H_{ES}^{\left( -1 \right)}+\varepsilon_{ES}^{\left( -1 \right)} & 0 & 0 & 0 & 0 \\ 0 & H_{SS}^{\left( -1 \right)}+\varepsilon_{SS}^{\left( -1 \right)} & 0 & 0 & 0 \\ 0 & 0 & H_{GS}^{\left( -1 \right)}+\varepsilon_{GS}^{\left( -1 \right)} & 0 & 0 \\ 0 & 0 & 0 & H_{ES}^{\left( 0 \right)}+\varepsilon_{ES}^{\left( 0 \right)} & 0 \\ 0 & 0 & 0 & 0 & H_{GS}^{\left( 0 \right)}+\varepsilon_{GS}^{\left( 0 \right)} \end{matrix} \right)$, (2)

where $H_{X}^{(Y)}$ and $\varepsilon_{X}^{(Y)}$ are the spin Hamiltonian and the energy of electronic state $X$ in charge state $Y$, respectively. The spin Hamiltonians of the excited state of the negatively charged NV centre can be written as

$H_{ES}^{\left( -1 \right)}=D_{ES}\left( S_{z}^{2}-\frac{2}{3} \right)+g_{e}\mu_{B}BS\boldsymbol{+}SA_{ES}I+P\left( I_{z}^{2}-\frac{2}{3} \right)-g_{14N}\mu_{N}BI$, (3)

where $S$ and $I$ are the electron and the ^14^N nuclear spin operators, $D_{ES}=1.42$ GHz is the zero-field-splitting and $A_{ES}$ is the hyperfine tensor with eigenvalues $A_{\perp}=27.45$ MHz and $A_{\parallel}=41.42$ MHz in the excited state and $P=-5.01$ MHz is the quadrupole splitting^7^. The ground state spin Hamiltonian in the same charge state can be written as

$H_{GS}^{\left( -1 \right)}=D_{GS}\left( S_{z}^{2}-\frac{2}{3} \right)+g_{e}\mu_{B}BS\boldsymbol{+}SA_{GS}I+P\left( I_{z}^{2}-\frac{2}{3} \right)-g_{14N}\mu_{N}BI$, (4)

where $D_{GS}$ and $A_{GS}$ are the zero-field splitting and the hyperfine tensor in the ground state^8^, respectively. For the singlet shelving state, we use the following expression

$H_{SS}^{\left( -1 \right)}=P\left( I_{z}^{2}-\frac{2}{3} \right)-g_{14N}\mu_{N}BI$. (5)

Additionally, we neglected the orbital momentum degrees of freedom in all electronic states, which is indispensable for understanding the spin selective non-radiative decay process, however, it only negligibly affects the spin dynamics of the electronic states^9^. As the Zeeman splitting of the doublet state suppresses hyperfine mixing between the electron and nuclear spins, the electron spin degrees of freedom of the spin-1/2 neutral charge state can be neglected at the magnetic field values considered in our experiments. Therefore, $H_{GS}^{\left( 0 \right)}=H_{ES}^{\left( 0 \right)}\approx H_{SS}^{\left( -1 \right)}$ was used in the simulations. Due to the opening of a large energy gap between the $m_{S} = +1$ and other electron spin sublevels in the ground and excited states of the negative charge state, only the most relevant $m_{S} = \left\{ 0,-1 \right\}$ spin states were included in our model. Furthermore, due to the non-unitary coupling between the electron spin states, the energy eigenstates $\varepsilon_{X}^{Y}$ could be set to zero without loss of generality.

**Supplementary Figure 7 |** **5-state optical excitation model.** The model includes five electronic states in two charge states [neutral NV(0) and negative NV(-1)]. Non-unitary transitions between the states are represented with coloured arrows, where $\Gamma_{xy}$ and $\sigma_{xy}$ are corresponding decay rates and photon absorption cross-sections, respectively. Dimensions of the electron and nuclear spin subspaces in the electronic states are given in brackets after the labels of the states, while the spin Hamiltonian of each state is provided in the text. NV(0) excited and ground states solely include nuclear spin degrees of freedom, similarly to the singlet shelving states in the negative charge state. The excited and the ground state of the NV(-1) include six states: the $m_{S} = \left\{ 0,-1 \right\}$ electron spin states and the triplet ^14^N nuclear spin states.

Transitions between the electronic states were modelled by Lindblad jump operators and related decay rates through the second term on the right-hand side of Supplementary Eq. (1). In the simulations, 13 Lindblad operators were used. Their action can be seen in Supplementary Figure 7 and the corresponding rates can be found in Supplementary Table 1. Decay rates were obtained from the literature^10–14^. Under the assumption that optical excitation rates depend linearly on the power in our model, we derived the formula

$\Gamma_{xy}= \sigma_{xy}P$, (6)

where $P$ corresponds to the laser power and $\sigma_{xy}$ is the photon absorption cross‑section of the transition between state $x$ and $y$.

**Supplementary Table 1. Rates and photon absorption cross‑sections used in the simulation.** Rates of $\sigma_{24}$, $\sigma_{74}$ and $\sigma_{53}$ are in the order of 1 MHz (see Supplementary Figure 10 for the actual values).

| **Rate / cross section** | **Value** | **Unit** |
| --- | --- | --- |
| $\sigma_{12}=\sigma_{67}$ | 13.2 | MHz/mW |
| $\sigma_{45}$ | 39.7 | MHz/mW |
| $\sigma_{24}=\sigma_{74}$ | Fitted | |
| $\sigma_{53}$ | Fitted | |
| $\Gamma_{21}=\Gamma_{76}$ | 77.0 | MHz |
| $\Gamma_{23}$ | 10.0 | MHz |
| $\Gamma_{73}$ | 91.0 | MHz |
| $\Gamma_{31}$ | 5.62 | MHz |
| $\Gamma_{36}$ | 1.41 | MHz |
| $\Gamma_{54}$ | 50.0 | MHz |

Neutral-to-negative and negative-to-neutral ionization rates are of crucial importance to understand the charge state dynamics and the related PDMR signal of the NV centre. Not all of these transitions have, however, been accurately characterized yet. Both intrinsic two‑photon absorption processes and charge carrier capture ionization processes are possible. The most likely two-photon ionization processes are depicted in Supplementary Figure 7. Accordingly, the negative-to-neutral charge state transition is characterized by rates ${}_{24}$ and ${}_{74}$ and the neutral-to-negative charge state transition is characterized by rate${}_{53}$. Rates ${}_{24}$, ${}_{74}$ and ${}_{53}$ are determined from fitting to the experimental curves.

Time‑dependent photoluminescence and photocurrent can be deduced from the occupation $n_{XS}^{(q)}$ of state $X$ in charge state $q$, which is calculated directly from the density matrix $\rho$, as well as the corresponding decay and excitation rates. To calculate the photoluminescence and the photoinduced electron and hole currents of the NV centre the following formulas were used:

$l^{(-1)}=\Gamma_{21}n_{ES,m_{S}=0}^{(-1)}+\Gamma_{76}n_{ES,m_{S}=\pm1}^{(-1)}$, (7)

$l^{(0)}=\Gamma_{54}n_{ES}^{(0)}$, (8)

$e=\sigma_{24}Pn_{ES,m_{S}=0}^{(-1)}+\sigma_{74}Pn_{ES,m_{S}=\pm1}^{(-1)}$, (9)

$h=\sigma_{53}Pn_{ES}^{(0)}$, (10)

where $l^{(X)}$ is the photon count in charge state $X$, and $e$ and $h$ are the electron and hole creation rates, respectively.

Our simulations directly model the pulse sequences used in the experiment to measure the electron spin and nuclear spin‑dependent PL traces, see Supplementary Figure 8 and Supplementary Figure 9 (see also main text). Microwave and radiofrequency pulses were modelled by projection operators acting on the spin state in the ground state of the negatively charged NV centre. We propagated the starting density matrix, corresponding to the pure state of $\left| \left. m_{S},m_{14N} \right\rangle_{-1, GS}= \right.\left| \left. 0,+1 \right\rangle\right.$, according to the pulse sequences used in the experiment until the PL trace in an optical excitation step is equal to the one in the preceding optical excitation step, i.e. a stationary solution of the time‑dependently driven system is obtained.

Our simulations described ideal MW and RF pulses and did not include PL background and currents originating from photoionization of other defects or dark currents. Disturbances and noise, however, may be present in the experiment and can decrease the signal-to-noise ratio. To compensate for this effect, we also fitted the amplitude of the PL curves to the experimental curves. From these fits, the effect of external disturbances can be estimated. We note that these disturbances do not alter the overall spin‑dependent dynamics of the decay that is defined by the model in Supplementary Figure 7 and the spin Hamiltonians in Supplementary Eq. (1)-(5).

The spin contrast of time‑dependent signals can be obtained from

$C\left[ S \right]=\frac{\int_{0}^{T} \left( S_{\left| \left. 0,1 \right\rangle\right.}\left( t \right)- S_{\left| \left. -1,1 \right\rangle\right.}\left( t \right) \right)dt}{\int_{0}^{T} S_{\left| \left. 0,1 \right\rangle\right.}\left( t \right)dt}$, (11)

where $T$ is the integration time and $S$ is the signal that can be either luminescence from the NV centre or photoinduced current.

**Supplementary Note 7: Theory of ESLAC transitions and modelling - comparison with experimental results**

Comparison of the experimental measurements and the theoretical curves for magnetic field values of 400 G, 440 G, and 500 G at moderate (0.34 mW) laser power are depicted in Supplementary Figure 8. The normalized PL traces of different initial states are shifted for visibility. As can be seen, the dynamics of the decay is well reproduced by our model for all magnetic field values considered in the experiment. These results validate the decay rates and the photon capture cross-section values used in the simulation at the low power limit. From the comparison of the PL traces and the required amplitude corrections we anticipate the presence of a 1-2 Gauss transverse magnetic field, whose contribution is suppressed at 400 G, see further discussion below, and 10-20% imperfection of the MW and RF pulses that may occur due to drift of the resonance frequency during averaging.

**Supplementary Figure 8 | Comparison of the experimental and theoretical PL traces for 0.34 mW laser power.** **a**, **b**, and **c** depict normalized PL traces obtained at 400 G, 440 G, and 500 G, respectively. Black, red, green, and orange curves show PL traces recorded after initialization into the states $\left| \left. 0,1 \right\rangle\right.$, $\left| \left. 0,0 \right\rangle\right.$, $\left| \left. -1,1 \right\rangle\right.$, and $\left| \left. -1,0 \right\rangle\right.$, respectively. For better visibility the curves correspond to $\left| \left. 0,0 \right\rangle\right.$, $\left| \left. -1,1 \right\rangle\right.$, and $\left| \left. -1,0 \right\rangle\right.$ initial states are shifted down with 0.05, 0.1 and 0.15, respectively.

**Supplementary Figure 9 | Comparison of the experimental and theoretical PL traces at 500 G.** **a**, **b**, **c**, and **d** depict normalized PL traces obtained for 0.34 mW, 1 mW, 3 mW, and 6 mW laser power, respectively. Black, red, green, and orange curves show PL traces recorded after initialization into the states $\left| \left. 0,1 \right\rangle\right.$, $\left| \left. 0,0 \right\rangle\right.$, $\left| \left. -1,1 \right\rangle\right.$, and $\left| \left. -1,0 \right\rangle\right.$, respectively. For better visibility the curves correspond to $\left| \left. 0,0 \right\rangle\right.$, $\left| \left. -1,1 \right\rangle\right.$, and $\left| \left. -1,0 \right\rangle\right.$ initial states are shifted down with 0.05, 0.1, and 0.15 respectively.

Supplementary Figure 9 shows a comparison of the PL traces at different laser powers at 500G combined with the theoretical curves that accurately reproduce the experimental time traces. Supplementary Figure 10 depicts the fitted ionization rates $\Gamma_{24}$ and $\Gamma_{74}$ and $\Gamma_{53}$ as a function of the applied laser power. It can be seen that both the negative-to-neutral and the neutral-to-negative rates saturate at high laser power, which may indicate major contributions of extrinsic effects to the charge state dynamics of the NV centre. Since our model solely includes NV related electronic states, it cannot explain the origin of these observations, however, we anticipate that inclusion of other defect levels in the environment of the NV centre may interfere with the charge state dynamics of the NV centre and explain the observed saturation of the rates. Additionally, at low laser powers, the neutral-to-negative ionization rate exceeds the rate of the reverse process. As a consequence, the negative charge state is populated at 93%. At 3 mW and above, however, the negative-to-neutral ionization process dominates, giving rise to a reduced population of 34% in the bright negative charge state.

**Supplementary Figure 10 | Laser power dependence of the ionization rates at 500G.** Green filled squares and maroon filled circles represent negative-to-neutral and neutral-to-negative charge transition rates, respectively.


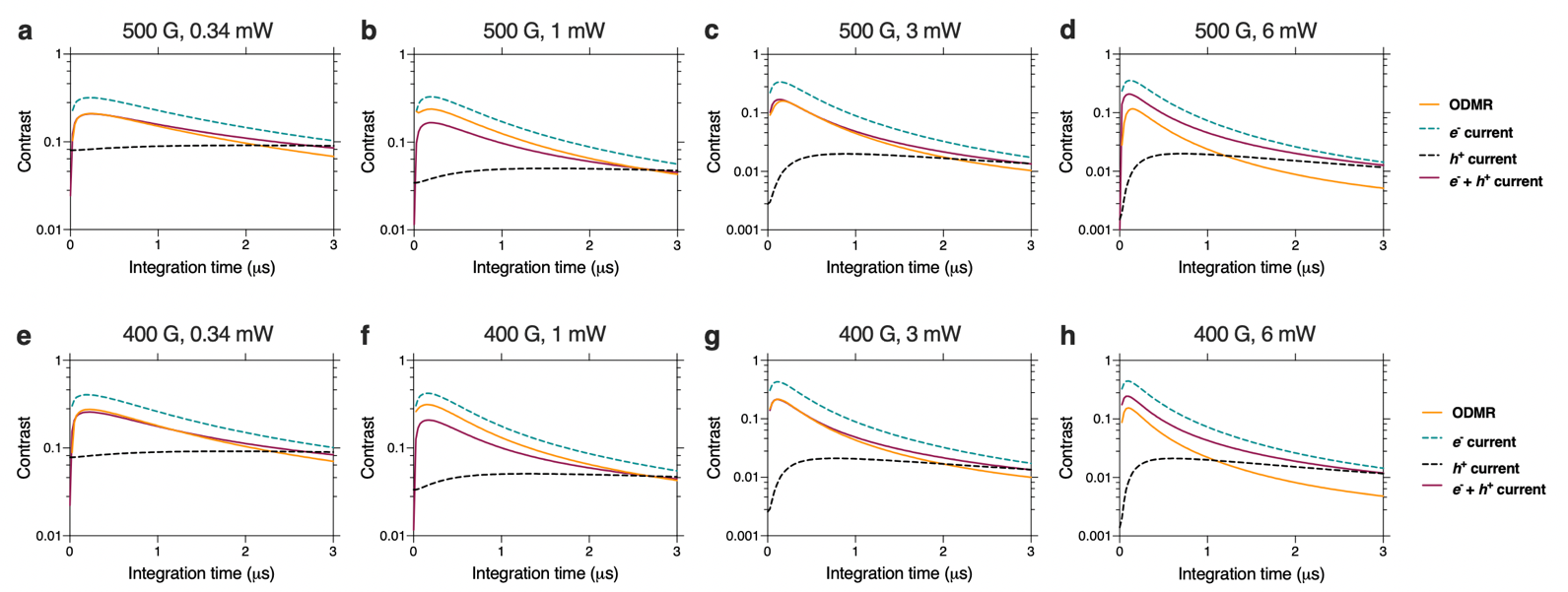


**Supplementary Figure 11 | Theoretical OMDR and PDMR contrasts.** **a**, **b**, **c**, and **d** show the ODMR (amber solid line), electron-only (teal dashed line), hole-only (black dashed line), and PDMR (purple solid line) contrasts versus the integration time for 0.34 mW, 1 mW, 3 mW, and 6 mW laser power at 500 G external magnetic field, respectively. **e**, **f**, **g**, and **h** show similar contrast curves at 0.34 mW, 1 mW, 3 mW, and 6 mW laser power at 400 G.

Supplementary Figure 11 depicts the theoretical ODMR and PDMR contrast curves as a function of the integration time $T$ for various laser powers at 400 G and 500 G magnetic fields. In addition to these curves, the contrast curve of the electron-only and the hole-only currents, obtained from our simulations, are depicted as well. The presented ODMR contrast curves match the experimental ones, following the good agreement between experimental and theoretical PL time traces depicted in Supplementary Figure 9. The predicted PDMR contrast curves incorporate three effects, i) contrast of the electron-only current, ii) contrast of the hole-only current, and iii) reduction of the contrast due to the background current measured to be 22%, 20.5%, 14%, and 9% of the NV current in our setup at 0.34 mW, 1 mW, 3 mW, and 6 mW, respectively. It can be seen that the electron-only and the hole-only contrast curves exhibit distinct integration time dependence. While electron-only curves are proportional to the ODMR curves, hole-only contrast curves show considerably smaller contrast, however, they are nearly independent on the integration time over the 3 $\mu$s interval depicted in Supplementary Figure 11. ODMR, as well as the electron-only contrast, vanish on the timescale of the reinitialization of the electron spin. On the other hand, the hole-only contrast vanishes on the timescale of a full charging-decharging cycle. Inclusion of the hole-only current has a negative effect on the PDMR contrast at short integration times, however, it can enhance the contrast at longer integration times.

The results depicted in Supplementary Figure 11 also reveal the excitation power dependence of the ODMR and PDMR contrast curves at two magnetic field values: at 500 G, which corresponds to the ESLAC, and at 400 G, which is sufficiently far from the ESLAC, such that the electron and nuclear spins are decoupled in all electronic states. As can be seen, the ODMR contrast is maximal at 1 mW laser power and exceeds the PDMR contrast, however, at any other laser power, we either find the ODMR and PDMR contrasts to be comparable or the PDMR contrast to be superior (see Supplementary Figure 11d and h for 6 mW). For the given integration time, the calculated PDMR contrast monotonously increases with the laser power, while the ODMR contrast decays for powers above 1 mW. The magnitude of this tendency is enhanced at longer integration times.

Next, we study the magnetic field dependence of the contrast obtained by using an RF π‑pulse to initialize the |0,0〉 state (RF contrast). Since the electron spin quantum number is not affected by the RF pulse, no contrast is expected in regular cases. On the other hand, hyperfine interaction can mix the |0,0〉 state with the |‑1,+1〉 state enhancing the non-radiative relaxation and reducing the PL intensity. Hyperfine interaction is most effective at the ESLAC, where the vanishing gap between the electron spin states enables efficient mixing with the nuclear spin states. Increased splitting of the electron spin states suppresses hyperfine coupling farther away from the ESLAC, thus the contrast depends considerably on the magnetic field. In Supplementary Figure 12 we depict the theoretical contrast obtained for the |0,0〉 state in ideal circumstances. As can be seen, the maximal contrast obtained for the |0,0〉 state may be as high as 34% close to the ESLAC. Note that the peak maximum appears at smaller magnetic field than the ESLAC (*D*_ES_ = 1420 MHz, *B*_ESLAC_ = 507 G), due to the strong hyperfine interaction that shifts the |‑1,+1〉 downward. The magnetic field dependence of the contrast follows a Lorentzian curve with a FWHM = 60 G.

When comparing with the experimental results depicted in Supplementary Figure 5 for 400 G and 500 G, we see that the theoretical maximal contrast is considerably higher that the measured ~20% contrast at 500 G. We attribute the difference to imperfections of the initiation and the read-out process as well as possible external noises. By using the perfectors fitted to the MW contrast curve previously, we obtain 20.4% contrast for the RF contrast, in line with the experimental observations. At 400 G the theoretical contrast (4%) falls below the experimental contrast (5%). We attribute this difference to the presence of a small residual transverse magnetic field that causes mixing between the |0,0> and the |‑1,0〉 state in addition to the hyperfine mixing between the |0,0〉 and the |-1,+1〉 states. This additional mixing enhances the RF contrast farther away the ESLAC.

**Supplementary Figure 12 | Simulated magnetic field dependence of the contrast obtained for the |0,0〉 state initialized by an RF π‑pulse.** The curve depicts the theoretical maximum of the contrast for ideal circumstances.

Modelling is carried out solely for the NV centre, however, we note that in real situation there are other charge carriers (for example, due to photoionization from other defects), carrier traps, and recombination centres that might need to be considered. Interactions of the electron or hole photocarriers, originating from the NV centre, with environmental defects may cause electron-hole recombination that in turn can affect the contrast. In particular, the electron-hole recombination via the defect states can lead to sub-linear dependence of the photocurrent as a function of the incoming photon rate and consequently to the spin contrast enhancement. In particular, sublinear dependence of the photocurrent as a function of the incoming photon rate is frequently observed for the situations where in addition to the centre surface recombination, multi centre recombination or trapping occur leading to such nonlinearity^15^. The quantitative description of the detected PDMR signal would require a combination of the presented NV centre excitation dynamics study with device simulation that is the subject of further studies.

**Supplementary Figure 13 | Photon and charge carrier emission rates at 500 G.** Green filled squares and maroon filled circles represent carrier and photon emission rates at different laser powers, respectively.

Background irradiation and leakage current are of major relevance in determining the signal-to-noise ratio in ODMR and PDMR measurements. Supplementary Figure 13 depicts the photon and carrier emission rates predicted by our simulations. As can be seen, the photon count rate shows a maximum at 1 mW, while the carrier emission rate keeps rising up to ~ 6 mW. The maximal photon emission rate at 1 mW and the enlarged population of the neutral charge state above 1 mW suggest that the signal-to-noise ratio is maximal at 1 mW in ODMR. On the other hand, maximal signal-to-noise ratio of the PDMR signal is expected at higher laser powers. This is supported by measurements revealing a reduction of the relative strength of the background current from 22% to 9% by going from 0.34 mW to 6 mW laser power. It should be noted that the power dependence of the carrier photo-emission rate presented here does not include effects mentioned above such as the recombination or trapping, which would be reflected in the carrier recombination lifetime. In that case, the carrier photo‑emission rate saturation would be determined by the carrier recombination lifetime value.

**Supplementary Figure 14 | Transverse magnetic field dependence of the contrast.** Red and green solid curves depict the transverse magnetic field dependence of the contrast as obtained exclusively from the PL signal of NV(-1) at 400 G and 500 G, respectively.

Finally, we examine a possible source of the reduced contrast at 500 G, namely spin mixing due to residual transverse magnetic fields. In Supplementary Figure 14, the theoretical transverse magnetic field dependence of the contrast is depicted as obtained from the PL emission signal of NV(-1) at two magnetic field values considered in the experiment. As can be seen, the contrast shows considerable dependence on the strength of the transverse magnetic field. This effect, however, is reduced as the magnetic field gets farther away from the ESLAC resonance point at ~500 G.

**Supplementary References**

1. Gulka, M. *et al.* Pulsed Photoelectric Coherent Manipulation and Detection of N-V Center Spins in Diamond. *Phys. Rev. Appl.* **7**, (2017).

2. Bourgeois, E., Gulka, M. & Nesladek, M. Photoelectric Detection and Quantum Readout of Nitrogen-Vacancy Center Spin States in Diamond. *Adv. Opt. Mater.* 1902132 (2020) doi:10.1002/adom.201902132.

3. Bube, R. H. Saturation of photocurrent with light intensity [7]. *Journal of Applied Physics* vol. 31 1301–1302 (1960).

4. Jacques, V. *et al.* Dynamic Polarization of Single Nuclear Spins by Optical Pumping of Nitrogen-Vacancy Color Centers in Diamond at Room Temperature. *Phys. Rev. Lett.* **102**, 057403 (2009).

5. Steiner, M., Neumann, P., Beck, J., Jelezko, F. & Wrachtrup, J. Universal enhancement of the optical readout fidelity of single electron spins at nitrogen-vacancy centers in diamond. *Phys. Rev. B - Condens. Matter Mater. Phys.* **81**, 035205 (2010).

6. Fischer, R., Jarmola, A., Kehayias, P. & Budker, D. Optical polarization of nuclear ensembles in diamond. *Phys. Rev. B - Condens. Matter Mater. Phys.* **87**, 125207 (2013).

7. Doherty, M. W. *et al.* The nitrogen-vacancy colour centre in diamond. *Phys. Reports, Vol. 528, Issue 1, p. 1-45.* **528**, 1–45 (2013).

8. Broadway, D. A. *et al.* Anticrossing Spin Dynamics of Diamond Nitrogen-Vacancy Centers and All-Optical Low-Frequency Magnetometry. *Phys. Rev. Appl.* **6**, 064001 (2016).

9. Reiserer, A. *et al.* Robust quantum-network memory using decoherence-protected subspaces of nuclear spins. *Phys. Rev. X* **6**, (2016).

10. Robledo, L., Bernien, H., Sar, T. van der & Hanson, R. Spin dynamics in the optical cycle of single nitrogen-vacancy centres in diamond. *New J. Phys.* **13**, 025013 (2011).

11. Siyushev, P. *et al.* Optically Controlled Switching of the Charge State of a Single Nitrogen-Vacancy Center in Diamond at Cryogenic Temperatures. *Phys. Rev. Lett.* **110**, 167402 (2013).

12. Thiering, G. & Gali, A. Theory of the optical spin-polarization loop of the nitrogen-vacancy center in diamond. *Phys. Rev. B* **98**, 085207 (2018).

13. Kalb, N., Humphreys, P. C., Slim, J. J. & Hanson, R. Dephasing mechanisms of diamond-based nuclear-spin memories for quantum networks. *Phys. Rev. A* **97**, (2018).

14. Bockstedte, M., Schütz, F., Garratt, T., Ivády, V. & Gali, A. Ab initio description of highly correlated states in defects for realizing quantum bits. *npj Quantum Mater.* **3**, 31 (2018).

15. Grimmeiss, H. G. Photoelectronic properties of semiconductors. By *Richard H. Bube* , Cambridge University Press, Cambridge 1992, 318 pp., paperback, £ 17.95, ISBN 0-521-40681-1. *Adv. Mater.* **5**, 65–66 (1993).
